# Trends in age‐specific incidence, mortality, and DALYs of female breast cancer from 1990 to 2021

**DOI:** 10.1002/agm2.12382

**Published:** 2024-12-25

**Authors:** Subhadra Priyadarshini, Kunja Bihari Panda

**Affiliations:** ^1^ Department of Statistics Utkal University Bhubaneswar Odisha India; ^2^ Department of Statistics Central University of Jharkhand Ranchi India

**Keywords:** age‐specific trends, breast cancer, DALYs, epidemiology, forecasting, incidence, mortality

## Abstract

**Objectives:**

Breast cancer is a leading cause of morbidity and mortality among women worldwide. This study aims to analyze the trends in breast cancer incidence, mortality, and disability‐adjusted life years (DALYs) across different age groups from 1990 to 2021, and to project the mortality rate for the next decade.

**Methods:**

Global breast cancer data were analyzed, focusing on three distinct age groups: 15–49 years, 50–69 years, and 70+ years. Joinpoint regression analysis was used to identify periods of significant changes in cancer rate trends (joinpoints). Age‐specific annual percent changes (APC) and average annual percent changes (AAPC) were calculated to identify trends over time. Additionally, an autoregressive integrated moving average (ARIMA) model was employed to forecast future mortality rates.

**Results:**

The overall incidence of breast cancer increased significantly with an AAPC of 1.6% from 1990 to 2021. The highest increase was observed in the 15–49 years age group (AAPC: 1.3%), while the 70+ years age group showed the lowest increase (AAPC: 0.2%). Mortality rates exhibited a complex pattern, with a modest overall increase (AAPC: 0.8%), a slight increase in the 15–49 years age group (AAPC: 0.4%), and decreases in both 50 and 69 years (AAPC: −0.4%) and 70+ years age groups (AAPC: –0.3%). DALY rates increased slightly overall (AAPC: 0.7%), primarily driven by the 15–49 years age group (AAPC: 0.4%), while the older age groups showed a declining trend (AAPC: −0.4%).

**Conclusion:**

Incidence rates are rising across all age groups, with the highest increase observed among younger women (15–49 years). In contrast, older age groups (50 + years) are experiencing improvements in mortality and DALYs. These findings underscore the need for targeted public health interventions, enhanced screening practices, and ongoing advancements in breast cancer treatment to address the evolving burden of this disease across different age groups.

## Graphical abstract

This study reveals significant age‐specific trends in breast cancer incidence, mortality, and disability‐adjusted life years (DALYs) from 1990 to 2021. Notably, the incidence rate (per 100,000) of breast cancer is rising, particularly among younger women (15–49 years), while older age groups are experiencing improvements in mortality and DALYs.

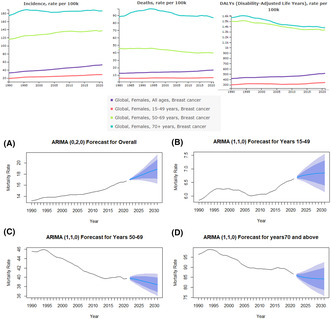

## 1 INTRODUCTION

### 1.1 Background

Breast cancer remains one of the most prevalent and impactful malignancies affecting women worldwide.^1^ Its burden is reflected not only in the number of new cases but also in the significant mortality and morbidity it causes.^2^, ^3^ As of the latest global cancer statistics, breast cancer accounts for approximately 24.5% of all cancer cases in women and remains the leading cause of cancer‐related deaths among women.^4^

The landscape of breast cancer has undergone significant changes over recent decades.^2^, ^5^, ^6^ Numerous initiatives have aimed at enhancing breast cancer outcomes, focusing on advanced screening methods like mammography and innovative treatment options such as targeted therapies and personalized medicine.^7^, ^8^, ^9^ These efforts have led to positive changes in breast cancer control, although the impact has not been the same across all age groups. Younger women often face unique challenges, including aggressive tumor biology^10^, ^11^ and delayed diagnosis, while older women may benefit more from advancements in treatment and early detection.^12^ Despite these advancements, the improvements have not been evenly distributed among age groups, highlighting the need for a detailed analysis of age‐specific trends.

Trend analysis has become a pivotal tool in epidemiology and public health to identify potential trends in specific quantities like counts, proportions, or rates over time in clinical oncology and basic cancer research.^13^ Joinpoint regression analysis is particularly useful for detecting points where a significant change in the trend occurs, providing a detailed understanding of the temporal dynamics of cancer metrics.^14^ Several studies have utilized joinpoint regression to analyze cancer trends, underscoring its versatility and effectiveness in cancer trend analysis and informing targeted interventions and policy decisions.^15^, ^16^, ^17^

Cancer data are an important record in public health as it is used to understand the burden and trends of cancer, assess the quality of cancer care, and measure progress in cancer control and prevention efforts. Breast cancer is an age‐related disease, with aging playing a significant role in its incidence and the efficacy of its treatment due to associated physical changes, including hormonal changes.^18^ Projecting trends in breast cancer incidence across different age groups can help identify age‐specific targets for incidence control policies.

### 1.2 Problem statement

Understanding the age‐specific trends in breast cancer incidence, mortality, and disability‐adjusted life years (DALYs) is crucial for developing effective public health strategies, improving clinical outcomes, and guiding future research priorities.

### 1.3 Objectives

This study aims to analyze the trends in breast cancer incidence, mortality, and DALYs from 1990 to 2021, with a focus on different age groups (15–49 years, 50–69 years, and 70+ years), and to project future mortality rates up to 2031.

## 2 MATERIALS AND METHODS

### 2.1 Study design

This is a retrospective study on longitudinal data examining trends in breast cancer incidence, mortality, and DALYs across different age groups from 1990 to 2021.

### 2.2 Data source

We obtained data from the Global Burden Disease (GBD), 2021 (available from https://ghdx.healthdata.org/gbd‐2021).^19^ This database contains worldwide health and demographic data compiled by GBD collaborators, offering a comprehensive evaluation of health impact related to 371 diseases and 88 risk factors across 204 countries and 811 sub‐national locations within 21 GBD regions. This source provided comprehensive records of breast cancer cases, deaths, and DALY calculations. The age‐standardized incidence, death, and DALYs for breast cancer were also compiled based on gender (female only), location (global), age (overall, 15–49 years, 50–69 years, and 70+ years), and year (from 1990 to 2021).

### 2.3 Statistical analysis

Joinpoint regression analysis was conducted to detect trends and identify significant changes in breast cancer metrics over time. The Joinpoint Regression Program, version 4.8.0.1, from the Statistical Methodology and Applications Branch, Surveillance Research Program, National Cancer Institute, Bethesda, MD,^20^ was used for this purpose. Segmentation analysis was carried out to identify joinpoints where the trend significantly changes. The annual percent change (APC) for each identified segment was calculated, and hypothesis tests were performed to determine the statistical significance of the APCs. The average annual percent change (AAPC) over the full range (1990–2021) was calculated to present the overall trend analysis for each age group and the overall population. 95% confidence intervals (CIs) for both APC and AAPC were determined to assess the precision of the estimates. The p‐values were calculated to test the null hypothesis that the APC or AAPC equals zero (indicating no significant trend), with a *p* < 0.05 indicating statistical significance. Autoregressive integrated moving average (ARIMA) time‐series model was used to forecast the future mortality rates, utilizing R version 4.3.3 software.

The details of the mathematical model are provided below.

#### 2.3.1 Joinpoint regression model

The joinpoint regression model for the observations, ($x_{1},y_{1}),\ldots ,\left(x_{n},y_{n}\right)$, with k + 1 segments, where $x_{1}\leq \ldots \leq x_{n}$, without loss of generality, may be written as.

(1) $$E\left(y|x\right)=\beta_{0}+\beta_{1}x+\delta_{1}{\left(x-\tau_{j}\right)}^{+}+\ldots ..+\delta_{k}{\left(x-\tau_{k}\right)}^{+}+\varepsilon_{i}$$

$$=\beta_{0}+\beta_{1}x+\sum_{j=1}^{k}\delta_{j}\left(x-\tau_{j}\right)$$

where, ${\left(x-\tau_{k}\right)}^{+}=\left\{\begin{array}{c} \left(x-\tau_{k}\right),x>\tau_{k}\;\\ \;0,\text{Other wise}\; \end{array}\right.$, $\delta_{k}=\beta_{\left(k+1\right)}-\beta_{k}\;\text{and}\;\varepsilon_{i}\approx N(0,\sigma^{2}$). $E\left(y|x\right)$: Expected value of the dependent variable y (e.g., incidence, mortality, or DALY rates) given the independent variable x (e.g., year). $\beta_{0}$: Intercept of the model, representing the initial level of y when x=0. $\beta_{1}$: Slope of the line before the first joinpoint, representing the rate of change in y before any significant trend change. $\delta_{j}$: Coefficient representing the change in slope at the $j^{\mathrm{th}}$ joinpoint. x: Independent variable, typically representing time (year). $\tau_{j}$: Location of the $j^{\mathrm{th}}$ joinpoint, where a significant change in trend occurs. $\epsilon_{i}$: Random error term, accounting for unexplained variability in the data.

#### 2.3.2 Annual percentage change (APC)

(2) $$\mathrm{APC}=e^{\beta_{1}+\delta_{1}+\delta_{2}+\ldots +\delta_{j-1}}\times 100$$

where, $\beta_{1}$: The slope of the regression line in the first segment of the data, representing the initial rate of change. $\delta_{j}$: The difference between the slope of the regression line in the $j^{\mathrm{th}}$ segment and the previous segment. j: Index representing the segment number (for joinpoints).

#### 2.3.3 Average annual percentage change (AAPC)

(3) $$\text{AAPC}=\exp\left(\sum_{i=1}^{k}\frac{w_{i}\beta_{i}}{w_{i}-1}\right)\times 100$$

where, $w_{i}$: Weight for the $i^{\mathrm{th}}$ segment, typically based on the length of the segment. $\beta_{i}$: Slope of the regression line in the $i^{\mathrm{th}}$ segment.

#### 2.3.4 Time‐series forecasting using ARIMA modeling

To forecast future cancer burden based on historical data, the ARIMA model is written as

(4) $$Y_{t}=c+\phi_{1}Y_{t-1}+\theta_{1}\epsilon_{t-1}+\epsilon_{t}$$

where, $Y_{t}$: Value of the time series at time t (e.g., cancer mortality at year t). c: Constant term, representing the mean level of the series if it is stationary. $\phi_{1}$: Autoregressive coefficient, representing the influence of the previous value $Y_{t-1}$ on the current value $Y_{t}$. $\theta_{1}$: Moving average coefficient, representing the influence of the past error $\epsilon_{t-1}$ on the current value $Y_{t}$. $\epsilon_{t}$: Error term at time t, representing the difference between the observed and predicted values.

### 2.4 Ethical considerations

As the study relied on publicly available de‐identified data, ethical approval was not required. All analyses were conducted following relevant data protection regulations and ethical guidelines for research involving secondary data analysis.

## 3 RESULTS

The global burden of female breast cancer has dramatically increased, with the number of new cases rising from 865,881 in 1990 to 2,082,737 in 2021. Table 1 provides a detailed overview of female breast cancer trends during this period, focusing on three key metrics: incidence (new cases), mortality (deaths), and DALYs across various age groups (all ages, 15–49 years, 50–69 years, and 70+ years).

**TABLE 1:** Overview of global trends in female breast cancer from 1990 to 2021.

		All	15–49 years	50–69 years	70+ years
Incidence	1990	32.7 (31.132, 34.019)	19.2 (18.401, 20.206)	116.03 (111.517, 120.479)	174.53 (158.711, 183.219)
	2021	52.97 (49.348, 56.590)	28.81 (26.844, 30.940)	137.21 (128.480, 147.576)	186.42 (159.541, 201.098)
Mortality	1990	13.24 (12.482, 13.914)	5.85 (5.506, 6.261)	45.55 (43.217, 48.071)	96.31 (87.439, 101.236)
	2021	16.8 (15.493, 17.985)	6.64 (6.173, 7.133)	39.79 (36.960, 42.725)	86.68 (74.026, 94.023)
DALYs	1990	416.80 (394.087, 440.780)	298.56 (280.003, 319.971)	1496.96 (1418.853, 1582.004)	1568.09 (1441.940, 1655.686)
	2021	515.13 (482.288, 548.694)	341.71 (317.737, 366.654)	1335.91 (1237.382, 1437.683)	1375.83 (1207.306, 1489.684)

*Note*: Data presented as rates per 100,000 women (95% confidence interval).

### 3.1 Incidence trends

Table 2 presents a detailed analysis of the trends in breast cancer incidence rates per 100,000 women across different age groups from 1990 to 2021, highlighting significant periods (segments) of change (joinpoints) in the APC and the overall AAPC. For the overall population, the incidence rate increased significantly across all segments, with notable periods of accelerated growth from 1990 to 1992 (APC: 1.9%), 1992 to 1995 (APC: 2.6%), 2002 to 2009 (APC: 1.8%), and 2014 to 2018 (APC: 1.8%). The overall AAPC for the period was 1.6%. For the 15–49 years age group, the highest growth occurred during 1990–1996 (APC: 2.9%), followed by smaller increases in subsequent periods, leading to an AAPC of 1.3%. In the 50–69 years age group, initial growth was moderate (1990–1995, APC: 1.5%) with fluctuations including a brief period of decline (2008–2014, APC: −0.1%), resulting in an AAPC of 0.6%. For the 70+ years age group, the incidence rate showed significant growth from 1990 to 1995 (APC: 1.6%) but fluctuated with periods of decline (2000–2004, APC: −0.6%) and a recent decline from 2018 to 2021 (APC: −0.3%), culminating in a modest AAPC of 0.2%. (Table 2).

**TABLE 2:** Age‐specific trends in female breast cancer incidence rate (per 100,000) from 1990 to 2021.

	Segment	Joinpoints	APC	CI of APC	*p*‐value	AAPC	CI of AAPC
Overall	1	1990–1992	1.86*	(1.62, 2.18)	<0.001	1.56*	(1.55, 1.58)
	2	1992–1995	2.57*	(2.26, 2.72)	<0.001		
	3	1995–2002	1.16*	(1.08, 1.23)	<0.001		
	4	2002–2009	1.77*	(1.70, 1.88)	<0.001		
	5	2009–2014	1.15*	(0.97, 1.27)	<0.001		
	6	2014–2018	1.83*	(1.69, 2.04)	<0.001		
	7	2018–2021	1.15*	(0.88, 1.34)	<0.001		
15–49 years	1	1990–1996	2.85*	(2.71, 3.00)	<0.001	1.33*	(1.30, 1.36)
	2	1996–2001	0.45*	(0.11, 0.69)	0.013		
	3	2001–2015	1.04*	(0.94, 1.11)	0.001		
	4	2015–2018	1.76*	(1.03, 1.95)	<0.001		
	5	2018–2021	0.75*	(0.18, 1.26)	0.026		
50–69 years	1	1990–1995	1.46*	(1.23, 1.71)	<0.001	0.56*	(0.54, 0.59)
	2	1995–2003	0.46*	(0.19, 0.60)	0.013		
	3	2003–2008	1.05*	(0.61, 1.45)	0.002		
	4	2008–2014	−0.07	(−0.41, 0.92)	0.324		
	5	2014–2021	0.25*	(0.12, 0.60)	0.003		
70+ years	1	1990–1995	1.63*	(1.47, 1.78)	<0.001	0.21*	(0.18, 0.23)
	2	1995–2000	−0.20	(−0.35, 0.64)	0.121		
	3	2000–2004	−0.60	(−0.89, 0.12)	0.084		
	4	2004–2018	0.20*	(0.17, 0.30)	0.001		
	5	2018–2021	−0.33*	(−0.77, −0.05)	0.025		

* Significant at 0.05 level.

**FIGURE 1:**
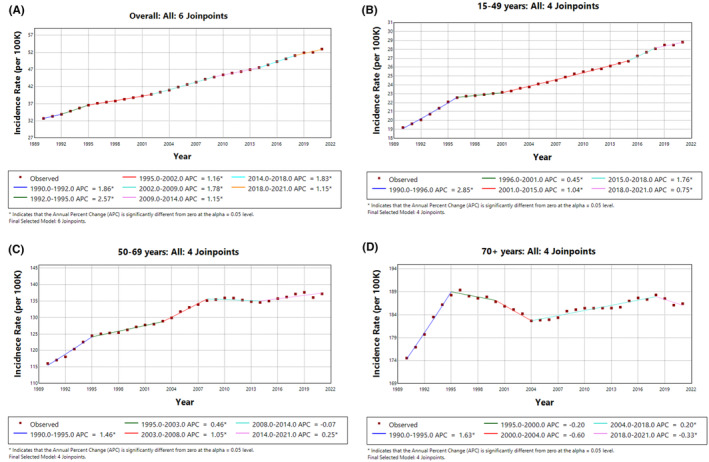
Trends in female breast cancer incidence rate (1990–2021). (A) Overall, (B) 15–49 years, (C) 50–69 years, and (D) 70+ years.

The trends in Figure 1 highlight significant increases in breast cancer incidence across all age groups, with variations in the rate and periods of growth. Overall, breast cancer incidence rates have been rising across all age groups from 1990 to 2021, with the highest increases observed in younger women (15–49 years).

### 3.2 Mortality trends

The data in Table 3 present the age‐specific trends in female breast cancer death rates (per 100,000) from 1990 to 2021, showing variations in APC and overall AAPC. For the overall population, breast cancer mortality rates increased significantly in several periods, particularly during 1990–1994 (APC: 1.1%), 2007–2014 (APC: 0.8%), and 2014–2018 (APC: 1.7%). Despite fluctuations, the overall AAPC for the period was 0.8%, indicating a moderate but steady rise in mortality rates. For the 15–49 years age group, mortality initially rose (1990–1996, APC: 1.3%) but saw periods of decline, particularly from 2003 to 2007 (APC: −0.8%). However, recent years (2012–2021) showed an increase (APC: 1.0%), resulting in an AAPC of 0.4%. The 50–69 years age group experienced an initial rise (1990–1994, APC: 0.3%), followed by a significant long‐term decline (1994–2013, APC: −0.7%), leading to an overall decrease in mortality (AAPC: −0.4%). For the 70+ years age group, mortality trends were more variable, with an initial increase (1990–1994, APC: 0.7%), followed by declines, notably from 2003 to 2006 (APC: −1.3%). The overall AAPC for this age group was −0.3%, indicating a slight decline in mortality rates over the period. (Table 3).

**TABLE 3:** Age‐specific trends in female breast cancer mortality rate (per 100 k) from 1990 to 2021.

	Segment	Joinpoints	APC	CI of APC	*p*‐value	AAPC	CI of AAPC
Overall	1	1990–1994	1.12*	(0.97, 1.30)	<0.001	0.78*	(0.76, 0.79)
	2	1994–1998	0.20	(0.00, 0.38)	0.053		
	3	1998–2003	0.56*	(0.46, 0.81)	0.003		
	4	2003–2007	0.13	(−0.12, 0.31)	0.168		
	5	2007–2014	0.85*	(0.74, 0.98)	<0.001		
	6	2014–2018	1.72*	(1.57, 1.99)	<0.001		
	7	2018–2021	0.89*	(0.59, 1.09)	<0.001		
15–49 years	1	1990–1996	1.25*	(1.08, 1.41)	<0.001	0.43*	(0.41, 0.45)
	2	1996–2003	−0.17	(−0.27, 0.03)	0.070		
	3	2003–2007	−0.84*	(−1.22, −0.55)	0.007		
	4	2007–2012	0.22	(−0.16, 0.67)	0.134		
	5	2012–2021	1.03*	(0.94, 1.18)	<0.001		
50–69 years	1	1990–1994	0.25	(0.04, 0.66)	0.090	−0.42*	(−0.44, −0.39)
	2	1994–2013	−0.75*	(−0.79, −0.72)	<0.001		
	3	2013–2021	0.05	(−0.07, 0.19)	0.328		
70+ years	1	1990–1994	0.67*	(0.53, 0.80)	<0.001	−0.34*	(−0.36, −0.32)
	2	1994–2003	−0.65*	(−0.70, −0.58)	0.002		
	3	2003–2006	−1.26*	(−1.38, −0.95)	<0.001		
	4	2006–2013	−0.14*	(−0.34, −0.05)	0.006		
	5	2013–2018	0.15*	(0.02, 0.37)	0.032		
	6	2018–2021	−1.11*	(−1.37, −0.84)	<0.001		

* Significant at 0.05 level.

**FIGURE 2:**
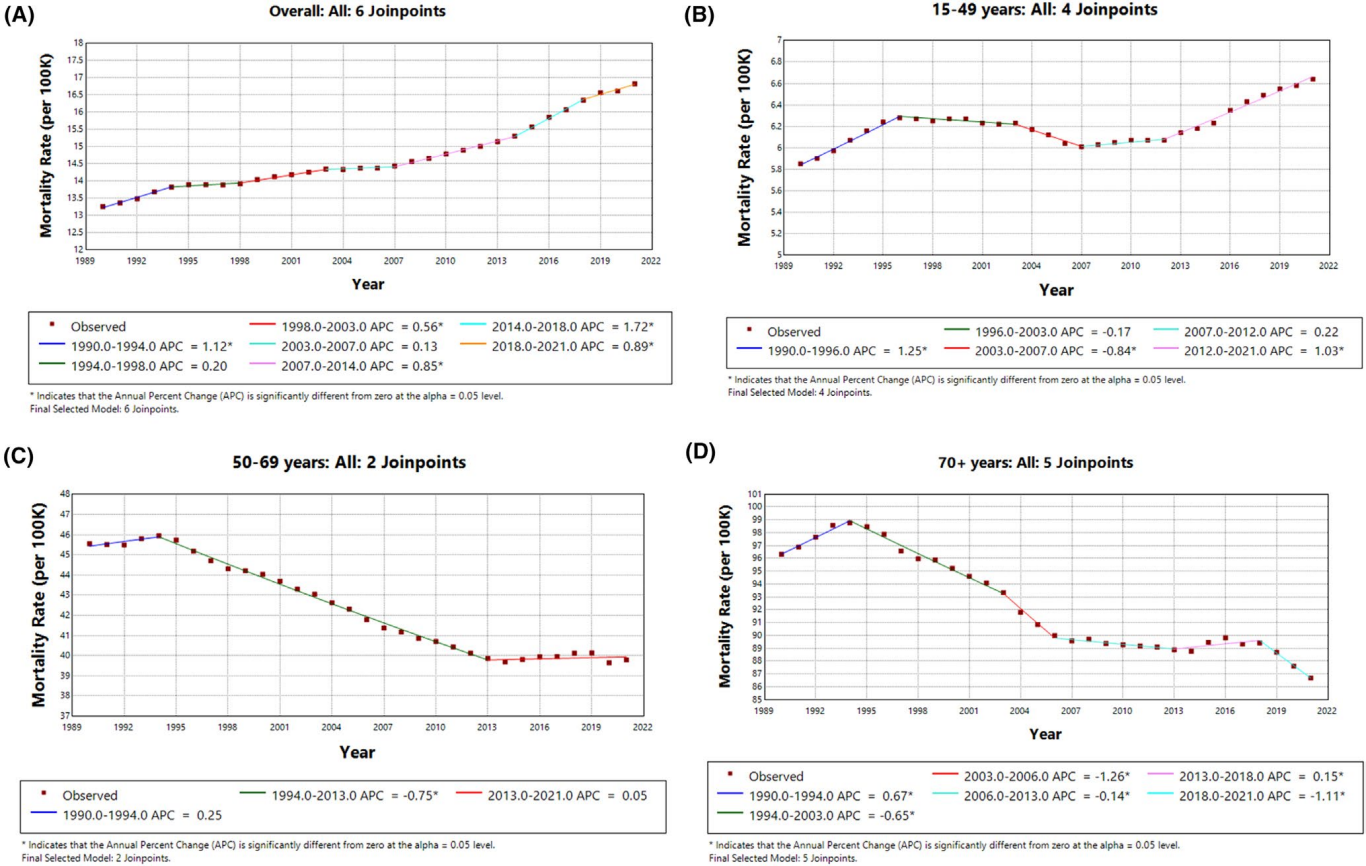
Trends in female breast cancer mortality rate (1990–2021). (A) Overall, (B) 15–49 years, (C) 50–69 years, and (D) 70+ years.

The trends in Figure 2 highlight varying patterns in breast cancer mortality across age groups, with significant declines in older age groups and more mixed trends in younger cohorts. This underscores the importance of continued efforts in breast cancer treatment and prevention, tailored to different age groups to address the observed mortality trends effectively.

### 3.3 DALY trends

The data in Table 4 illustrate age‐specific trends in DALY rates for female breast cancer (per 100,000) from 1990 to 2021, showcasing changes in APC and overall AAPC. For the overall population, the DALYs increased significantly during multiple periods, notably during 1990–1995 (APC: 1.0%), 2007–2013 (APC: 0.6%), and 2013–2018 (APC: 1.4%). Despite periods of minimal change or nonsignificant variations, the overall AAPC for the period was 0.7%, indicating a consistent rise in the burden of breast cancer. For the 15–49 years age group, the DALYs initially rose during 1990–1996 (APC: 1.2%), followed by a decline during 2003–2007 (APC: −0.8%). However, recent years (2013–2021) saw increases, leading to an overall AAPC of 0.44%. In the 50–69 years age group, after a small initial rise (1990–1994, APC: 0.2%), the DALYs showed significant long‐term declines, particularly from 1994 to 2003 (APC: −0.5%) and 2003 to 2013 (APC: −0.7%), resulting in an overall decrease in DALYs (AAPC: −0.35%). For the 70+ years age group, the trends were more variable, with an initial increase (1990–1994, APC: 0.6%) followed by significant declines from 1994 to 2002 (APC: −0.7%) and 2002 to 2006 (APC: −1.3%), and a minor recent decline from 2018 to 2021 (APC: −0.6%), culminating in an overall AAPC of −0.42%. (Table 4).

**TABLE 4:** Age‐specific trends in female breast cancer DALY rates (per 100,000) from 1990 to 2021.

	Segment	Joinpoints	APC	CI of APC	*p*‐value	AAPC	CI of AAPC
Overall	1	1990–1995	0.96*	(0.88, 1.08)	<0.001	0.69*	(0.67, 0.70)
	2	1995–1998	0.06	(−0.08, 0.29)	0.341		
	3	1998–2003	0.58*	(0.49, 0.81)	0.001		
	4	2003–2007	0.11	(−0.09, 0.26)	0.242		
	5	2007–2013	0.58*	(0.48, 0.77)	<0.001		
	6	2013–2018	1.38*	(1.28, 1.62)	<0.001		
	7	2018–2021	0.87*	(0.57, 1.05)	<0.001		
15–49 years	1	1990–1996	1.22*	(1.08, 1.34)	<0.001	0.44*	(0.41, 0.46)
	2	1996–2003	−0.15	(−0.24, 0.15)	0.083		
	3	2003–2007	−0.78*	(−1.09, −0.19)	0.018		
	4	2007–2013	0.31	(−0.73, 0.49)	0.088		
	5	2013–2018	1.29*	(0.36, 1.65)	0.003		
	6	2018–2021	0.74*	(0.3, 1.09)	0.009		
50–69 years	1	1990–1994	0.22	(0.00, 0.59)	0.052	−0.35*	(−0.37, −0.33)
	2	1994–2003	−0.51*	(−0.6, −0.32)	0.004		
	3	2003–2013	−0.67*	(−0.94, −0.24)	0.03		
	4	2013–2021	−0.07	(−0.18, 0.06)	0.223		
70+ years	1	1990–1994	0.65*	(0.52, 0.79)	<0.001	−0.42*	(−0.44, −0.41)
	2	1994–2002	−0.70*	(−0.76, −0.64)	<0.001		
	3	2002–2006	−1.29*	(−1.53, −1.12)	<0.001		
	4	2006–2014	−0.39*	(−0.54, −0.31)	<0.001		
	5	2014–2018	0.03	(−0.15, 0.22)	0.693		
	6	2018–2021	−0.62*	(−0.94, −0.44)	<0.001		

* Significant at 0.05 level.

**FIGURE 3:**
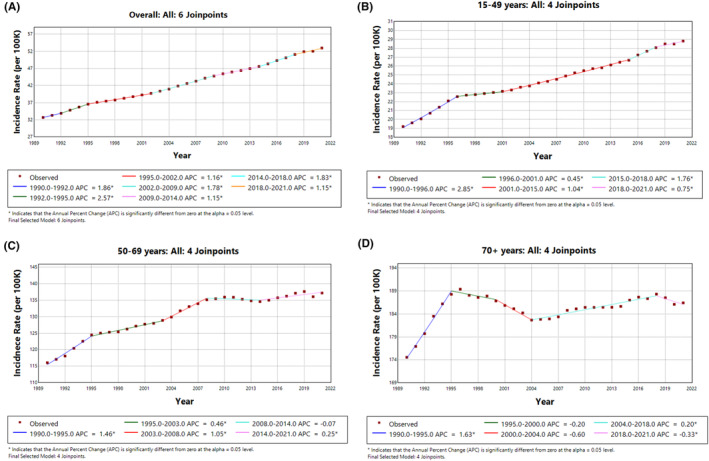
Trends in Female Breast Cancer DALY Rates (1990–2021). (A) Overall, (B) 15–49 years, (C) 50–69 years, and (D) 70+ years.

The trends in Figure 3 indicate a general increase in the burden of breast cancer as measured by DALYs in the overall population and especially in the younger age group (15–49 years), while older age groups (50–69 years and 70+ years) experienced reductions. The varying trends highlight the importance of targeted interventions and health care strategies to address the distinct needs of different age groups in managing the burden of breast cancer.

### 3.4 Forecast analysis for mortality rates

**FIGURE 4:**
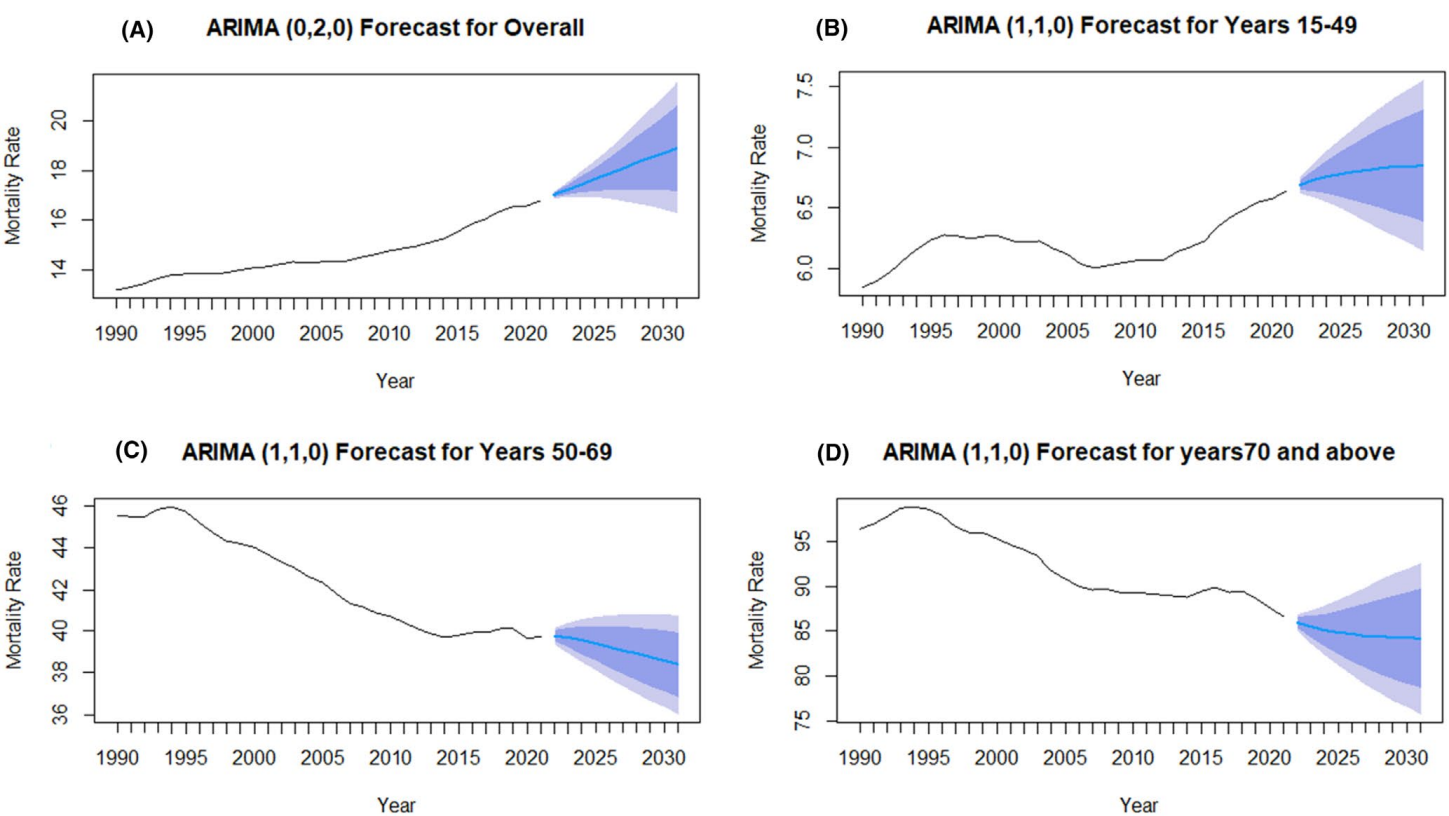
Future forecast of age‐specific female breast cancer mortality rate (per 100,000) for 2031. (A) Overall, (B) 15–49 years, (C) 50–69 years, and (D) 70+ years.

The ARIMA model was utilized to project future trends in breast cancer mortality rates up to the year 2031, as illustrated in Figure 4. The forecast analysis suggests a continued modest increase in overall mortality rates if current trends persist. Specifically, the 15–49 years age group is expected to see a further slight rise in mortality, reflecting the ongoing challenges in early diagnosis and treatment in younger populations. Conversely, the 50–69 years and 70+ years age groups are projected to maintain their declining mortality trends, driven by sustained improvements in treatment and possibly enhanced preventive measures. Detailed statistical outputs and projections for these trends are provided in the Supplementary File—Data S1.

## 4 DISCUSSION

The present study provides a detailed analysis of breast cancer trends from 1990 to 2021, focusing on incidence, mortality, and DALYs across different age groups. The findings reveal important insights into the changing landscape of breast cancer epidemiology, highlighting both progress and ongoing challenges.

The overall increase in incidence (AAPC: 1.6%) aligns with previous studies indicating a global rise in breast cancer cases,^21^ potentially due to lifestyle changes, reproductive factors, and increased screening and detection.^4^ The significant increase in breast cancer incidence, particularly among younger women (15–49 years), is a notable finding (AAPC = 1.3%). This rise could be attributed to several factors, including changes in reproductive patterns, lifestyle factors such as diet and physical activity, and increased exposure to risk factors like hormonal contraceptives and environmental toxins.^18^, ^22^, ^23^ The increase in incidence underscores the need for targeted awareness and prevention strategies tailored to younger women.

Despite the rise in incidence, breast cancer mortality rates have exhibited a more complex trend. The overall mortality rate has increased modestly (AAPC: 0.8%), but there is a notable age‐specific variation. The slight increase in mortality in the 15–49 years age group (AAPC: 0.4%) is concerning. Younger women typically face more aggressive breast cancer subtypes, such as triple‐negative and HER2‐positive cancers, which are associated with poorer prognoses and higher mortality rates.^10^, ^11^ This aggressiveness is compounded by the fact that younger women have limited screening opportunities and challenges in accessing timely and effective treatment^12^, ^18^, ^23^, ^24^, ^25^ compared to their older counterparts. Current breast cancer screening guidelines are primarily designed for women aged 50 and above, based on the higher incidence of the disease in this age group.^12^, ^26^ As a result, younger women are often not eligible for routine mammography, leading to later‐stage diagnoses and more advanced disease at the time of detection. The lower awareness of breast cancer risk among younger women, combined with the dense breast tissue that makes mammograms less effective, further delays diagnosis.

The rising mortality among younger women, despite advancements in early detection and treatment, underscores the need for more tailored interventions. The aggressive nature of breast cancer in this age group necessitates early and prompt treatment, which may not be fully realized due to delayed diagnosis. Furthermore, younger women often face unique psychosocial challenges, including concerns about fertility, career disruptions, and the impact of a cancer diagnosis on family life, which can influence their treatment choices and adherence to therapy.^27^, ^28^ These factors may contribute to poorer outcomes and higher mortality rates in younger women, despite overall improvements in breast cancer management.

In contrast, the older age groups (50–69 years and 70+ years) have seen a decrease in mortality rates (with AAPCs of −0.4% and −0.3%, respectively). These declines can be attributed to the widespread implementation of mammography screening in older patients and advances in therapies, including targeted therapies and personalized medicine, which have likely contributed to these positive trends.^24^, ^29^, ^30^ Mortality projection (Figure 4) also reveals an expected rise in mortality rates among younger women (ages 15–49) and a continued decline among older women (ages 50–69 and 70+). Understanding these trends is essential for anticipating future public health needs and formulating effective breast cancer prevention and treatment strategies.

Finally, the trends in DALYs highlight the disparities in disease burden across age groups. The overall increase in DALYs (AAPC: 0.7%) reflects a growing burden of breast cancer, particularly among younger women (15–49 years, AAPC: 0.4%), where both mortality and morbidity have significantly impacted their productive years and overall life expectancy.^31^ This trend indicates that despite advancements in treatment and survival, the disease's long‐term impact remains substantial for younger women.^28^, ^32^ Conversely, older women (AAPC: −0.4%), who are more likely to be part of regular screening programs, experience earlier detection and more effective treatment, resulting in lower DALY figures.^33^

### 4.1 Implications for public health and clinical practice

The results of this study underscore the importance of age‐specific strategies in breast cancer prevention, screening, and treatment. For younger women, there is a critical need to enhance awareness and accessibility of early screening programs, as well as to develop and implement interventions that address the aggressive nature of the disease in this group. Additionally, health care providers should consider the long‐term impact of breast cancer treatment on the quality of life for younger survivors and provide appropriate support services.

For older women, the declining trends in mortality and DALYs suggest that current screening and treatment strategies are effective, but ongoing efforts are necessary to sustain these improvements. The emphasis should be on maintaining high screening coverage and ensuring that older patients have access to the latest treatment modalities. Moreover, the management of comorbidities and the overall well‐being of older breast cancer patients should remain a priority.

### 4.2 Strengths and limitations

One of the key strengths of this study is its comprehensive analysis of age‐specific trends in breast cancer incidence, mortality, and DALYs over an extended period. The use of joinpoint regression and ARIMA modeling provided robust insights into the temporal dynamics of breast cancer burden, enabling the identification of significant shifts in trends and the projection of future mortality rates.

However, this study also has several limitations, including potential data quality issues, changes in diagnostic criteria over time, and incomplete data from some registries. These factors may influence the observed trends and it should be considered when interpreting the results.

## 5 CONCLUSION

This study provides a comprehensive analysis of breast cancer trends over three decades, highlighting significant age‐specific variations in incidence, mortality, and DALYs. While there have been significant improvements in reducing mortality and the overall burden of breast cancer in older women, the rising incidence and associated burden in younger women (15–49 years) highlight the need for ongoing efforts in prevention, early detection, and equitable access to advanced treatments. These trends underscore the critical need for age‐targeted public health interventions and policies. This study emphasizes the importance of adapting breast cancer prevention and control strategies to address the evolving burden of the disease across different age groups, ultimately aiming to reduce the overall impact of breast cancer on women's health globally.

## AUTHOR CONTRIBUTIONS

*Conception and design of study*: S.P. *Acquisition of data*: S.P. *Data analysis and/or interpretation*: S.P. *Drafting*: S.P and K.B.P. *Critical revision of manuscript*: KBP. *Approval of final version of manuscript*: all authors.

## 6 CONFLICT OF INTEREST STATEMENT

The authors have no conflicts of interest to declare.

## Supporting information


**Data S1:** Supporting Information.

## Data Availability

This study is conducted using data from the GBD 2021 database, which is available publicly and does not include personally identifiable information.
