# Supplementary material for: Trends in age‐specific incidence, mortality, and DALYs of female breast cancer from 1990 to 2021
Source: Aging Med (Milton). 2024 Dec 25;7(6):770–80. doi: 10.1002/agm2.12382 (PMC11702493; doi:10.1002/agm2.12382)
Supplement: Supplementary file 1 — Data S1: Supporting Information. [file AGM2-7-770-s001.docx]

**Supplementary File**

| **Sl No.** | **Contents** | **Page No.** |
| --- | --- | --- |
| 1 | Supplementary Table 1. Forecast of female breast cancer mortality rate (per 100,000) for Overall population | 2 |
| 2 | Supplementary Table 2. Forecast of female breast cancer mortality rate (per 100,000) for 15-49 years age group | 2 |
| 3 | Supplementary Table 3. Forecast of female breast cancer mortality rate (per 100,000) for 50-69 years age group | 3 |
| 4 | Supplementary Table 4: Forecast of female breast cancer mortality rate (per 100,000) for 70+ years age group | 3 |

Supplementary Table 1. Forecast of female breast cancer mortality rate (per 100,000) for Overall population

| Year | Point forecast | Lower 80% | Upper 80% | Lower 95% | Upper 95% |
| --- | --- | --- | --- | --- | --- |
| 2022 | 17.020 | 16.933 | 17.107 | 16.887 | 17.153 |
| 2023 | 17.230 | 17.036 | 17.424 | 16.933 | 17.527 |
| 2024 | 17.440 | 17.115 | 17.765 | 16.942 | 17.938 |
| 2025 | 17.650 | 17.174 | 18.126 | 16.922 | 18.378 |
| 2026 | 17.860 | 17.215 | 18.505 | 16.874 | 18.846 |
| 2027 | 18.070 | 17.240 | 18.900 | 16.801 | 19.339 |
| 2028 | 18.280 | 17.251 | 19.309 | 16.706 | 19.854 |
| 2029 | 18.490 | 17.248 | 19.732 | 16.590 | 20.390 |
| 2030 | 18.700 | 17.232 | 20.168 | 16.455 | 20.945 |
| 2031 | 18.910 | 17.204 | 20.616 | 16.300 | 21.520 |

Supplementary Table 2. Forecast of female breast cancer mortality rate (per 100,000) for 15-49 years age group

| Year | Point forecast | Lower 80% | Upper 80% | Lower 95% | Upper 95% |
| --- | --- | --- | --- | --- | --- |
| 2022 | 6.688 | 6.645 | 6.731 | 6.622 | 6.754 |
| 2023 | 6.726 | 6.637 | 6.814 | 6.590 | 6.861 |
| 2024 | 6.756 | 6.619 | 6.893 | 6.546 | 6.966 |
| 2025 | 6.780 | 6.594 | 6.967 | 6.495 | 7.065 |
| 2026 | 6.799 | 6.564 | 7.035 | 6.439 | 7.160 |
| 2027 | 6.815 | 6.531 | 7.098 | 6.381 | 7.248 |
| 2028 | 6.827 | 6.497 | 7.156 | 6.323 | 7.331 |
| 2029 | 6.836 | 6.462 | 7.211 | 6.264 | 7.409 |
| 2030 | 6.844 | 6.427 | 7.261 | 6.206 | 7.482 |
| 2031 | 6.850 | 6.392 | 7.309 | 6.149 | 7.551 |

Supplementary Table 3. Forecast of female breast cancer mortality rate (per 100,000) for 50-69 years age group

| Year | Point forecast | Lower 80% | Upper 80% | Lower 95% | Upper 95% |
| --- | --- | --- | --- | --- | --- |
| 2022 | 39.782 | 39.517 | 40.047 | 39.377 | 40.187 |
| 2023 | 39.693 | 39.214 | 40.173 | 38.961 | 40.426 |
| 2024 | 39.564 | 38.895 | 40.234 | 38.541 | 40.588 |
| 2025 | 39.415 | 38.578 | 40.251 | 38.135 | 40.694 |
| 2026 | 39.255 | 38.270 | 40.239 | 37.749 | 40.761 |
| 2027 | 39.089 | 37.972 | 40.207 | 37.380 | 40.798 |
| 2028 | 38.921 | 37.683 | 40.159 | 37.028 | 40.814 |
| 2029 | 38.752 | 37.403 | 40.100 | 36.689 | 40.814 |
| 2030 | 38.582 | 37.130 | 40.033 | 36.362 | 40.801 |
| 2031 | 38.411 | 36.864 | 39.958 | 36.045 | 40.778 |

Supplementary Table 4: Forecast of female breast cancer mortality rate (per 100,000) for 70+ years age group

| Year | Point forecast | Lower 80% | Upper 80% | Lower 95% | Upper 95% |
| --- | --- | --- | --- | --- | --- |
| 2022 | 86.007 | 85.416 | 86.597 | 85.104 | 86.910 |
| 2023 | 85.509 | 84.324 | 86.693 | 83.697 | 87.321 |
| 2024 | 85.140 | 83.344 | 86.937 | 82.393 | 87.887 |
| 2025 | 84.868 | 82.470 | 87.266 | 81.200 | 88.536 |
| 2026 | 84.666 | 81.688 | 87.644 | 80.111 | 89.221 |
| 2027 | 84.517 | 80.986 | 88.048 | 79.117 | 89.917 |
| 2028 | 84.407 | 80.352 | 88.462 | 78.205 | 90.608 |
| 2029 | 84.325 | 79.775 | 88.875 | 77.366 | 91.284 |
| 2030 | 84.265 | 79.246 | 89.284 | 76.589 | 91.941 |
| 2031 | 84.220 | 78.757 | 89.683 | 75.865 | 92.575 |
